# The Regulatory Mechanism of MiR-122 in the Antiviral Immunity of *Larimichthys crocea* Kidney Cells

**DOI:** 10.3390/ani16213489

**Published:** 2026-11-04

**Authors:** Yuyang Wang, Lifang Wen, Wenjin He, Jia Fu, Panyang Zheng, Yuxuan Chen, Mingshan Song, Baoying Guo, Weifeng Wang

**Affiliations:** 1 College of Marine Science and Technology, Zhejiang Ocean University, Zhoushan 316022, China; daiwa_scarlet@126.com (Y.W.); hewenjin0225@zjou.edu.cn (W.H.); fujia0616@zjou.edu.cn (J.F.); 1104057448cyx@gmail.com (Y.C.);; 2 National Engineering Research Center for Marine Aquaculture Facilities, Zhejiang Ocean University, Zhoushan 316022, China; luvmsfyy@126.com

**Keywords:** *Larimichthys crocea*, miR-122, transcriptome analysis, slc40a1, prkci

## Abstract

*Larimichthys crocea* is an important marine aquaculture fish in China, but its farming industry has long been threatened by viral diseases. MicroRNAs (miRNAs) are short non-coding RNAs that regulate gene expression post-transcriptionally, typically by inducing mRNA degradation or translational repression. And they have emerged as essential players in orchestrating antiviral-related cellular responses in fish. In the present study, the large yellow croaker kidney cell line (PCK) was used as an in vitro model. Through transfection of miR-122 mimics and inhibitors, combined with poly(I:C) immune stimulation, transcriptome, dual-luciferase reporter assays and qPCR validation were employed to systematically investigate the immune regulatory mechanism of miR-122. Transcriptome analysis revealed that 1995 and 1577 genes were differentially expressed after miR-122 overexpression and inhibition, respectively, and they were significantly enriched in pathways such as ECM–receptor interaction, focal adhesion, PPAR signaling, and TGF-β signaling. In these DEGs, *prkci* and *slc40a1* were confirmed as direct target genes of miR-122 in large yellow croaker via target gene prediction and dual-luciferase reporter assays. Furthermore, miR-122 overexpression significantly inhibited poly(I:C)-induced superoxide dismutase (SOD) activity, suggesting its involvement in the regulation of oxidative stress. These results support miR-122-responsive transcriptional and redox changes in poly(I:C)-stimulated PCK cells, mediated by targeting prkci and slc40a1 and by affecting SOD-mediated redox balance. This study provides new insights into the miRNA-mediated immune regulatory network in teleosts and offers potential molecular targets for disease-resistant breeding and the development of disease prevention and control strategies in large yellow croaker.

## Simple Summary

Large yellow croaker is an important marine aquaculture fish in China, but viral diseases cause severe losses to its farming. The kidney is important for fish immune defense. In this study, researchers used large yellow croaker kidney cells (PCK cells) grown in the laboratory and stimulated them with poly(I:C), a synthetic mimic of viral double-stranded RNA. They increased or decreased the level of a small RNA called miR-122 to study its role in immune-related cellular responses. Changing miR-122 affected many genes involved in cell adhesion, metabolism, and immune signaling. Reporter assays supported sequence-specific interactions of miR-122 with the 3′-untranslated regions of prkci and slc40a1. MiR-122 overexpression reduced SOD activity relative to the poly(I:C) and negative-control groups. These findings describe miR-122-associated responses under poly(I:C)-stimulated in vitro conditions. These findings improve our understanding of how small RNAs regulate fish immunity and may help identify targets for disease-resistant breeding or antiviral strategies.

## 1. Introduction

The large yellow croaker (*Larimichthys crocea*) is a vital marine aquaculture species in China, with annual production exceeding 250,000 metric tons. Its production is dominated by bay-cage farming, which accounts for more than 95% of total production, although deep-sea cages, offshore farming vessels, and enclosure systems are also being developed. The rapid expansion of high-density aquaculture has increased the risk of bacterial, viral, and parasitic diseases, posing important challenges to the sustainable development of the industry [1,2]. Viral disease outbreaks cause substantial economic losses [3,4]. As the innate immune system constitutes the first line of defense for fish against viruses, a comprehensive understanding of its regulatory mechanisms is essential for disease prevention and control.

MicroRNAs (miRNAs) are a class of endogenous, single-stranded, non-coding small RNAs, typically 21–26 nucleotides in length [5]. Although they lack protein-coding potential, miRNAs modulate gene expression at the post-transcriptional stage and are therefore involved in a wide range of biological activities [6]. In host antiviral immune responses, intracellular miRNAs act mainly through three routes: they can target host receptors required for viral entry, regulate intracellular signaling networks, and modulate cytokines linked to viral replication or immunity [7,8,9]. In fish, miRNAs have likewise been demonstrated to contribute to antiviral defense, inflammatory regulation, and immune homeostasis [10,11,12]. The antiviral effects of miRNAs are context-dependent and may result from direct interactions with viral genomes or from regulation of host factors involved in viral entry, replication, innate immune signaling, and cytokine production. Consequently, changes in miRNA abundance can either restrict or facilitate viral infection depending on the virus, host cell type, and stage of infection. These properties make miRNAs important regulators of host–virus interactions and potential molecular targets for studying antiviral responses. Accordingly, miRNA-focused investigation offers a practical perspective for deciphering the immune reactions of the large yellow croaker to virus infection.

In mammals, miR-122 predominantly regulates hepatic metabolism [13] and acts as an essential modulator in human innate antiviral immunity [14]. In hepatitis C virus (HCV) infection, miR-122 is an essential host factor for viral replication, and it promotes viral proliferation by binding to two sites in the 5′ untranslated region of the HCV genome [15]. miR-122 plays a restrictive role against hepatitis B virus (HBV), inhibiting viral replication, while HBV in turn suppresses miR-122 expression [16]. In addition, miR-122 can regulate type I interferon expression by targeting SOCS1 [17]. In teleost fish, the functions of miR-122 are more diversified. MIR122HG is the host gene of miR-122 in Miichthys miiuy. It suppresses antiviral innate immunity by targeting MAVS [18]. In addition, in *Epinephelus coioides*, miR-122 participates in regulating immune response against bacterial infection by targeting IL-15 [19]. In *Ctenopharyngodon idella*, fatty hepatocyte-derived exosomal miR-122 reduces the immune function and antioxidant defense capacity of kidney (CIK) cells [20]. In *Danio rerio*, the miR-122 expression level changed after *Vibrio harveyi* infection and lipopolysaccharide stimulation, and immune-related genes were upregulated after transfection [21]. Nonetheless, the direct downstream targets and the global regulatory network of miR-122 in *L. crocea* remain largely unexplored.

In a previous study [22], we stimulated large yellow croaker (*Larimichthys crocea*) with poly(I:C) and performed miRNA sequencing. The results revealed that miR-122 expression changed markedly across tissues and exhibited clear immune-responsive characteristics. These findings suggest that miR-122 may play an important role in the immune system of this fish. However, research to date has mainly focused on miR-122 expression patterns, and systematic transcriptomic analyses of its immunoregulatory function are still lacking. Poly(I:C), a synthetic analog of viral double-stranded RNA, is an ideal model for studying antiviral responses in fish [23], and transcriptome sequencing can comprehensively reveal the immune-related genes and signaling pathways regulated by miR-122. Therefore, this study used an in vitro PCK cell model to investigate the effects of miR-122 modulation on cellular responses under poly(I:C) stimulation. Because all RNA-seq groups were exposed to poly(I:C), the present design was intended to characterize miR-122-associated responses in a stimulated cellular context rather than to determine whether these effects are specific to poly(I:C) stimulation or also occur under basal conditions. Four experimental groups were established by transfecting miR-122 mimics, mimics NC, miR-122 inhibitor, and inhibitor NC, comprising 12 samples in total. Total RNA was extracted from each group for transcriptome sequencing. By integrating bioinformatics prediction, transcriptome data analysis, and dual-luciferase reporter assays, prkci and slc40a1 were identified and further confirmed as target genes of miR-122. We also demonstrated that miR-122 regulates SOD activity in PCK cells. These results will contribute to understanding the role of miRNAs in regulating immune responses during infection in fish.

## 2. Materials and Methods

### 2.1. Cell Culture and Transfection

Large yellow croaker kidney cells (PCK cells; CCTCC catalog no. GDC0173) were obtained from the Cell Bank of the China Center for Type Culture Collection (CCTCC), Wuhan University, China. According to the CCTCC records, the cells are adherent, negative for bacterial and fungal contamination, and negative for mycoplasma. The cells were cultured in MEM containing 10% fetal bovine serum (FBS) at 28 °C with 5% CO_2_. The cells were passaged at a 1:2 ratio every 2–3 days. Cell identity was recorded according to the CCTCC catalogue information; no additional genetic authentication was performed in this study. PCK cells were plated into 6-well plates and, once they reached 80% confluence, were separately transfected with miR-122 mimics, mimics negative-control sequence (mimics NC), miR-122 inhibitor, or inhibitor negative-control sequence (inhibitor NC). The final concentration of the transfected oligonucleotides was 50 nM. Each condition was performed in three biological replicates. At 24 h post-transfection, poly(I:C) was added at a final concentration of 10 μg/mL, and the cells were cultured for an additional 24 h. Transfections were carried out using Hieff Trans^®^ Liposomal Transfection Reagent (YEASEN Biotech, Shanghai, China) together with 50 nM of the respective oligonucleotides (GenePharma, Shanghai, China). The mimics NC and inhibitor NC were non-targeting negative-control sequences supplied by GenePharma. The oligonucleotide sequences and their labels are listed in Table 1. HEK293T cells were cultured in high-glucose DMEM medium supplemented with 10% fetal bovine serum (FBS, Sijiqing Biotechnology, Hangzhou, China) and 1% penicillin-streptomycin (Pricella, Wuhan, China).

### 2.2. Library Preparation and Sequencing

The concentration and purity of total RNA were assessed using a NanoDrop spectrophotometer, and RNA integrity was evaluated using an Agilent 5300 Bioanalyzer (Agilent Technologies, Inc., Santa Clara, CA, USA). RNA samples with a total amount of approximately 1 μg, a concentration of ≥20 ng/μL, and an RQN > 4.5 were used for library preparation. RNA purification, reverse transcription, library preparation, and sequencing were conducted by Shanghai Majorbio Bio-pharm Biotechnology Co., Ltd. (Shanghai, China) in strict accordance with the manufacturer’s protocols. For the preparation of the RNA-seq transcriptome library, 1 µg of total RNA was processed using the Illumina^®^ Stranded mRNA Prep, Ligation kit (San Diego, CA, USA). Briefly, messenger RNA was enriched through polyA selection with oligo(dT) magnetic beads, followed by initial fragmentation using a fragmentation buffer. Double-stranded cDNA was synthesized using random hexamer primers. During second-strand synthesis, dUTP was incorporated, and the dUTP-containing second strand was removed using uracil-DNA glycosylase to preserve strand-specific information. The cDNA fragments then underwent end repair, phosphorylation, A-tailing, and adapter ligation. The resulting cDNA fragments, approximately 300–400 bp in size, were selected using magnetic beads and amplified by PCR for 10–15 cycles. Following quantification with a Qubit 4.0 fluorometer, the sequencing libraries were ultimately run on either the NovaSeq X Plus platform (PE150) utilizing the NovaSeq Reagent Kit, or the DNBSEQ-T7 platform (PE150) employing the DNBSEQ-T7RS Reagent Kit (FCL PE150) version 3.0.

### 2.3. Quality Control and Read Mapping

Raw paired-end reads were processed using fastp (version 0.23.4) [24] with default settings for trimming and quality filtering. The resulting clean reads were mapped to the Larimichthys crocea reference genome (species: Larimichthys crocea; assembly accession: GCF_000972845.2) with HISAT2 (version 2.2.1) [25] in orientation mode. The reference genome was downloaded from the NCBI Genome database (https://www.ncbi.nlm.nih.gov/datasets/genome/GCF_000972845.2/) (accessed on 20 September 2025). The aligned reads from each sample were subsequently assembled using StringTie (version 2.2.1) [26] following a reference-guided strategy.

### 2.4. Differential Expression Analysis and Functional Enrichment

For differential expression analysis, gene-level expression abundance was estimated using RSEM (version 1.3.3) [27], and TPM (transcripts per million reads) values were used to represent gene expression abundance; TPM values were used only for descriptive expression analysis or visualization. Differentially expressed genes (DEGs) between groups were identified using DESeq2 (version 1.42.0) [28] (https://bioconductor.org/packages/stats/bioc/DESeq2/) (accessed on 25 September 2025) based on raw count data. DESeq2 uses a negative binomial distribution to model count data. Count data were normalized using the size-factor normalization method. *p*-values were adjusted for multiple testing using the BH (fdr correction with Benjamini–Hochberg) procedure. Genes with an FDR < 0.05 and |log_2_FC| > 0 were considered differentially expressed genes (DEGs). Furthermore, GO and KEGG enrichment analyses were performed to detect DEGs significantly overrepresented in specific GO terms and metabolic pathways. Enrichment significance was defined as a Bonferroni-corrected *p*-value < 0.05 against the whole-transcriptome background. GO analysis was conducted with Goatools (version 1.4.4), and KEGG pathway analysis was performed using the Python (version 3.13.7) scipy library.

### 2.5. Go and Kegg Annotation Analysis

The GO database was used to classify genes according to the biological processes (BP), cellular components (CC), and molecular functions (MF) in which they are involved, and GO annotation was performed on the differentially expressed genes. The KEGG database was used to categorize genes according to the pathways or functions in which they participate, and KEGG annotation was also performed on the differentially expressed genes.

### 2.6. Plasmid Construction

Genomic DNA isolated from large yellow croaker cells served as the template for PCR amplification of the 3′-UTR regions of *slc40a1* and *prkci*. The primers were designed with SacI and XhoI restriction sites at their termini. After double digestion with SacI and XhoI, the resulting fragments were inserted into the multiple cloning site located downstream of the firefly luciferase gene in the pmirGLO dual-luciferase reporter vector, which had been linearized with the same two enzymes. The ligation products were then introduced into competent *E. coli* JM109 (Promega, Madison, WI, USA) via transformation, and recombinant clones were selected on ampicillin-containing agar plates.

### 2.7. Sod Activity Assay

PCK cells were transfected with miR-122 mimics, mimics NC, and an equal volume of PBS, respectively. After 24 h, poly(I:C) was added at a final concentration of 10 μg/mL and the cells were further cultured for 24 h. Cells were collected, washed twice with pre-cooled PBS, and SOD activity was measured using a SOD activity assay kit (WST-1 method) from Beijing Solarbio Science & Technology Co., Ltd. (Beijing, China) according to the manufacturer’s instructions. All experiments were performed with three independent biological replicates.

### 2.8. Dual-Luciferase Reporter Assay

HEK-293T cells were seeded into 24-well plates. Upon reaching 70% confluence, cells were co-transfected with pmirGLO plasmids harboring either the wild-type (WT) or mutant (MUT) 3′-UTR sequence of slc40a1 or prkci, together with miR-122 mimics or the corresponding mimics NC. WT and MUT constructs were tested in parallel, and an empty pmirGLO vector was included as a reference control. After 48 h of transfection, the cells were harvested, washed with PBS, and luciferase activities were measured using the Dual-Luciferase^®^ Reporter Assay System (Promega) strictly following the manufacturer’s instructions. Firefly luciferase activity was first normalized to Renilla luciferase activity, and the resulting Firefly/Renilla ratios were then normalized to the empty pmirGLO vector NC group, which was set to 1. Three independent biological experiments were performed, each with three technical replicate wells. Luciferase activity was analyzed using two-way ANOVA, with reporter construct (WT, MUT, or empty pmirGLO vector) and treatment (miR-122 mimics or mimics NC) as the two factors. Tukey’s multiple-comparisons test was used for pairwise comparisons, including comparisons between miR-122 mimics and mimics NC within each reporter construct and comparisons among the WT, MUT, and empty pmirGLO vector constructs under the mimics NC condition.

### 2.9. Real-Time Quantitative Pcr

Total RNA was extracted from relevant samples. Subsequently, first-strand cDNA was synthesized using the PrimeScript™ RT reagent Kit with gDNA Eraser (Takara, Japan), strictly following the manufacturer’s instructions. The expression levels of immune-related genes were determined by qRT-PCR. Primers were designed using Primer 6.0 software (Table 2), and *β*-actin was used as an internal control for normalization. The thermal cycling program for qRT-PCR was 95 °C for 30 s, followed by 40 cycles of 95 °C for 5 s and 60 °C for 30 s. Data analysis was performed using the relative CT (2^−^ΔΔCt) method, with each sample undergoing three independent replicates. The results are presented as mean ± SD.

### 2.10. Statistical Analysis

Statistical analyses were conducted using GraphPad Prism 10.1 (GraphPad Software, San Diego, CA, USA). For cell experiments performed using six-well plates, each independent six-well plate was considered one independent biological replicate and the experimental unit, with three technical replicates included for each biological replicate. Two-group comparisons were analyzed using two-tailed unpaired Student’s *t*-tests. Experiments involving more than two groups were analyzed using one-way analysis of variance (one-way ANOVA), followed by Tukey’s multiple-comparisons test. For the dual-luciferase reporter assays, two-way analysis of variance (two-way ANOVA) was performed, with reporter construct (WT, MUT, or empty pmirGLO vector) and treatment (miR-122 mimics or mimics NC) as the two factors. Tukey’s multiple-comparisons test was used for the prespecified pairwise comparisons, including comparisons between miR-122 mimics and mimics NC within each reporter construct and comparisons between the WT and MUT constructs under the mimics NC condition. Three independent co-transfection experiments were analyzed. Technical replicate wells within each experiment were averaged before inferential testing. Values are presented as mean ± standard deviation (mean ± SD), and *p* < 0.05 was considered statistically significant.

## 3. Results

### 3.1. Description of Rna-Sequencing Datasets

In this RNA-seq analysis, twelve *L. crocea* kidney cell (PCK) samples were utilized. After rigorous quality filtering, a high-confidence dataset of 121 Gb was obtained. The proportions of bases with Q20 and Q30 quality scores reached 98% and 94% (Table S1), respectively, and the overall GC content was 50%. All clean reads have been deposited in the NCBI Sequence Read Archive (SRA) under accession number PRJNA1501314.

### 3.2. Statistics of Differentially Expressed Genes (Degs)

**Figure 1:**
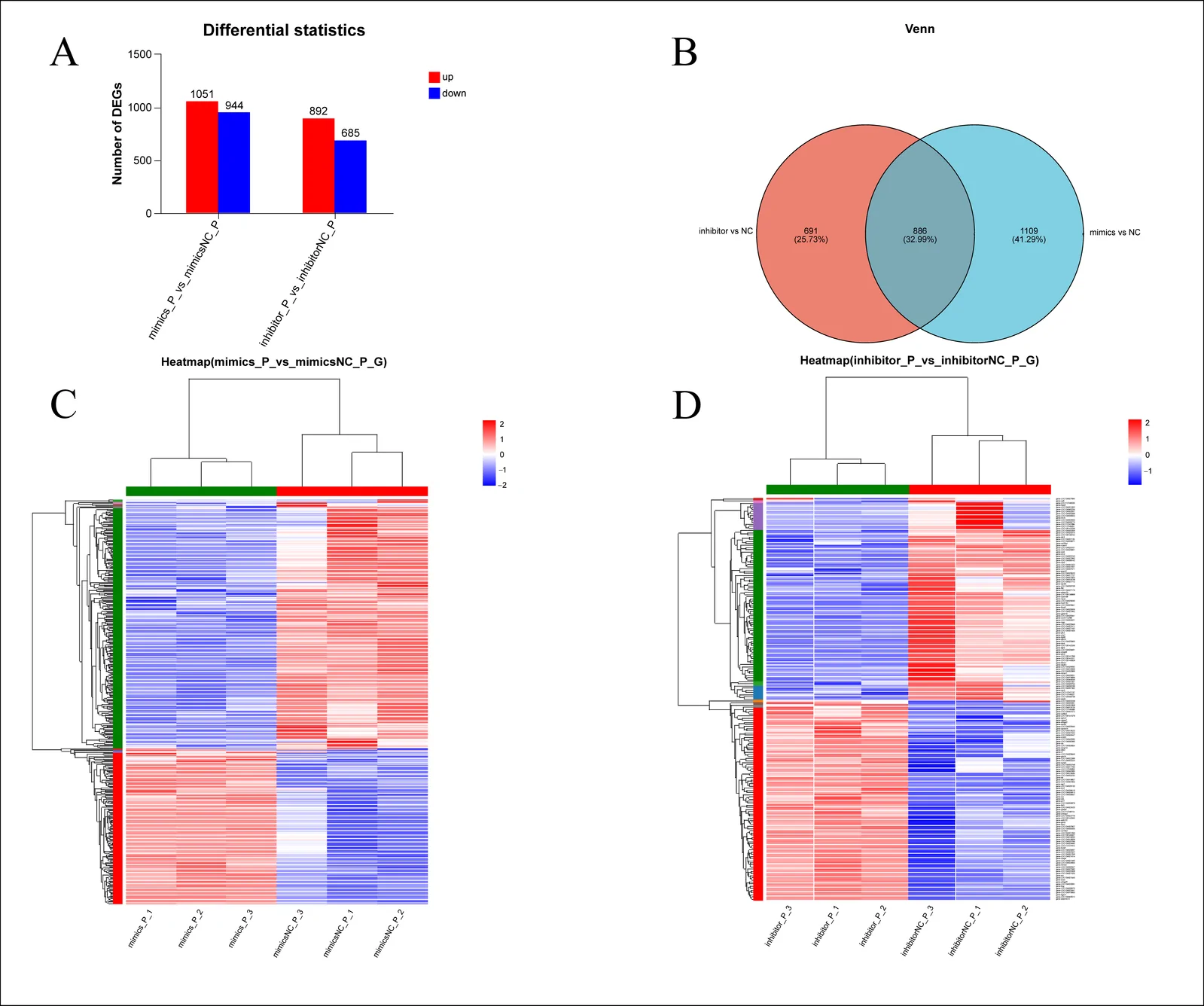
Differential gene expression analysis in PCK cells upon treatment with miR-122 mimics, mimics NC, inhibitor, and inhibitor NC. (**A**) Differentially expressed genes (DEGs) identified in mimics vs. NC and inhibitor vs. inhibitor NC comparisons (|log_2_FoldChange| > 0, adjusted *p* < 0.05). Upregulated and downregulated DEGs are indicated by red and blue bars, respectively. (**B**) Venn diagram comparing DEGs between the two comparisons. (**C**,**D**) Heatmaps of DEGs for mimics vs. NC and inhibitor vs. inhibitor NC.

The comparison between the mimics group and its negative control (NC) revealed 1995 differentially expressed genes (DEGs), of which 1051 were upregulated and 944 were downregulated (Figure 1A). In parallel, the inhibitor group versus its NC comparison produced 1577 DEGs, comprising 892 upregulated and 685 downregulated transcripts (Figure 1A). Heatmaps of these DEGs showed pronounced expression differences in both the mimics and inhibitor groups (Figure 1C,D). Notably, a total of 108 genes showed co-regulation in the two experimental comparisons (Figure 1B).

### 3.3. Functional Enrichment Analysis of Differentially Expressed Genes

**Figure 2:**
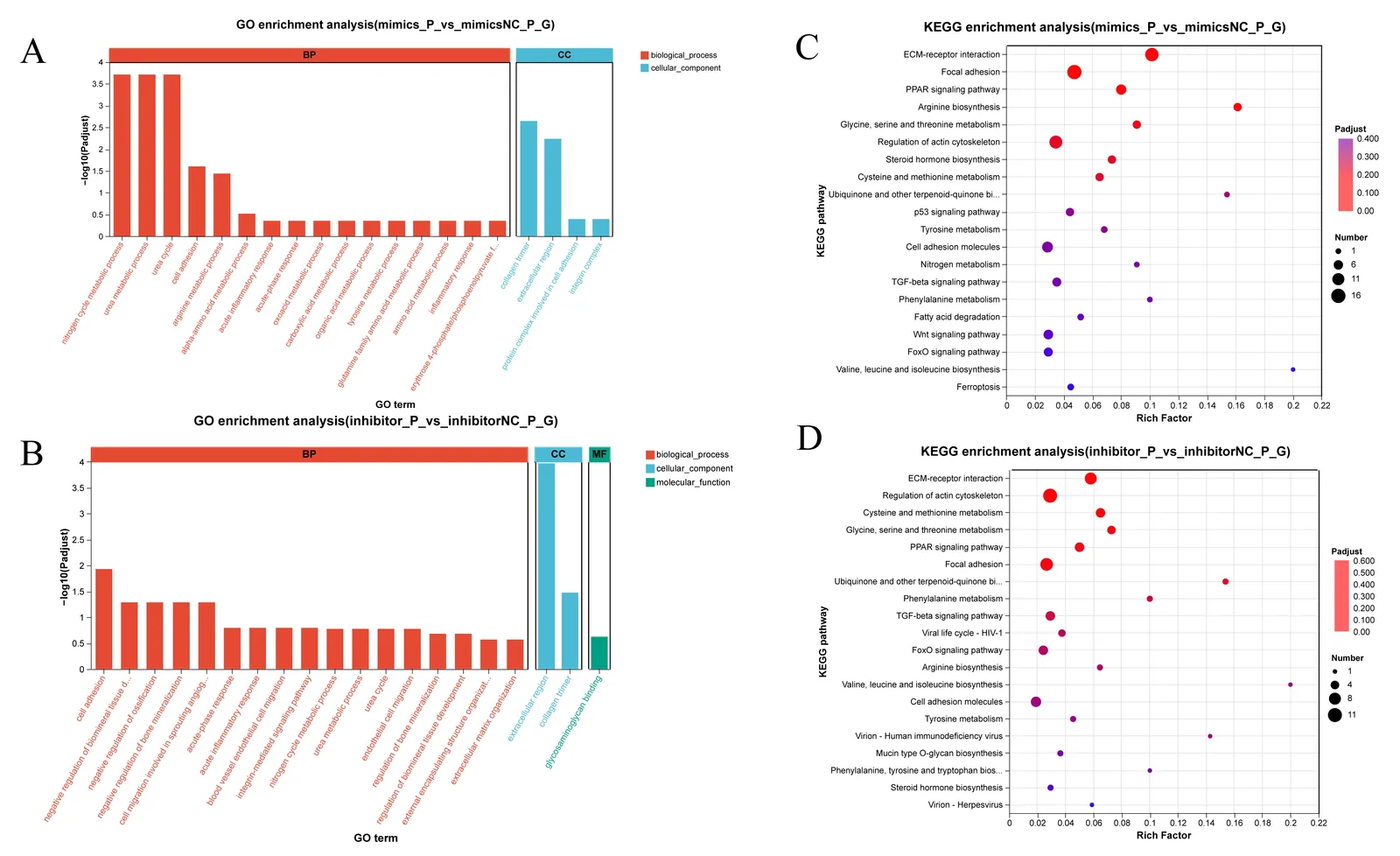
GO and KEGG enrichment analysis. (**A**) Top 20 GO terms in the miR-122 mimics versus mimics NC comparison. (**B**) Top 20 GO terms in the miR-122 inhibitor versus inhibitor NC comparison. (**C**) Significantly enriched KEGG pathways in the miR-122 mimics versus mimics NC comparison. (**D**) Significantly enriched KEGG pathways in the miR-122 inhibitor versus inhibitor NC comparison.

GO enrichment analysis was performed to classify the differentially expressed genes (DEGs) in the mimics and inhibitor groups of PCK cells. The GO analysis encompassed the domains of Cellular Component, Molecular Function, and Biological Process. Comparisons were made between the mimics and NC groups, and between the inhibitor and inhibitor NC groups (Figure 2). In the mimics group, the majority of DEGs were mainly enriched in the Cellular Component and Biological Process categories (Figure 2A). Within the Cellular Component category, the collagen trimer (GO:0005581) and extracellular region (GO:0005576) were the dominant subclasses. Regarding the Biological Process category, the most abundant subclasses were the nitrogen cycle metabolic process (GO:0071941) and urea metabolic process (GO:0019627). In the inhibitor group, the majority of DEGs were enriched across all three categories (Figure 2B). Within the Cellular Component category, the most significant categories were the extracellular region (GO:0005576) and collagen trimer (GO:0005581). Within the Biological Process category, cell adhesion (GO:0007155), negative regulation of biomineral tissue development (GO:0070168), and negative regulation of ossification (GO:0030279) were identified as the most abundant subclasses. Within the Molecular Function category, the predominant DEGs were categorized under glycosaminoglycan binding (GO:0005539).

According to the KEGG pathway enrichment analysis, a total of 99 pathways were significantly upregulated in the mimics group, featuring ECM–receptor interaction, focal adhesion, PPAR signaling, and regulation of actin cytoskeleton, among others (Figure 2C). In contrast, the inhibitor group exhibited 89 significantly enriched pathways, which included TGF-beta signaling, ECM–receptor interaction, Cysteine and methionine metabolism, and endocytosis (Figure 2D).

### 3.4. Qpcr Validation

**Figure 3:**
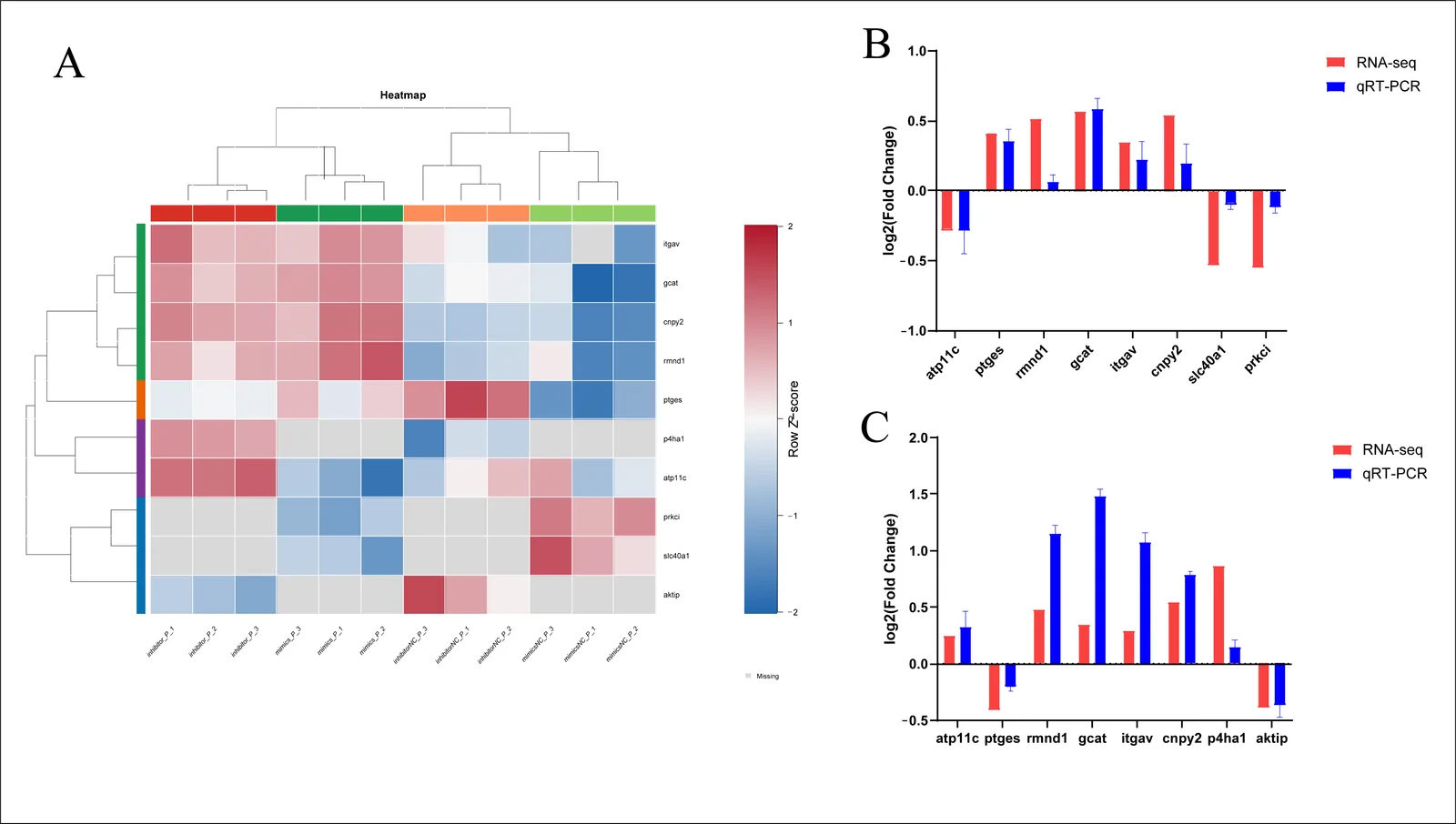
Validation of DEGs using quantitative real-time PCR. The experiments were performed using three independent biological replicates (*n* = 3). (**A**) Expression levels of ten selected genes. (**B**) Results of qRT-PCR and RNA-seq for eight selected genes in the mimics versus mimics NC comparison. (**C**) Results of qRT-PCR and RNA-seq for eight selected genes in the inhibitor versus inhibitor NC comparison.

To verify the reliability of the transcriptome data in this study, ten DEGs were selected in total for qPCR validation: eight genes from the mimics versus mimics NC comparison and eight genes from the inhibitor versus inhibitor NC comparison, with six genes shared between the two comparisons. The selected genes were *atp11c*, *ptges*, *rmnd1*, *gcat*, *itgav*, *cnpy2*, *slc40a1*, *prkci*, *p4ha1*, and *aktip* (Figure 3A; Table 3A,B). The qPCR and RNA-seq results showed concordant expression trends within each comparison (Figure 3B,C), supporting the reliability of the transcriptome results.

### 3.5. Mir-122 Regulates Sod Activity in Pck Cells

**Figure 4:**
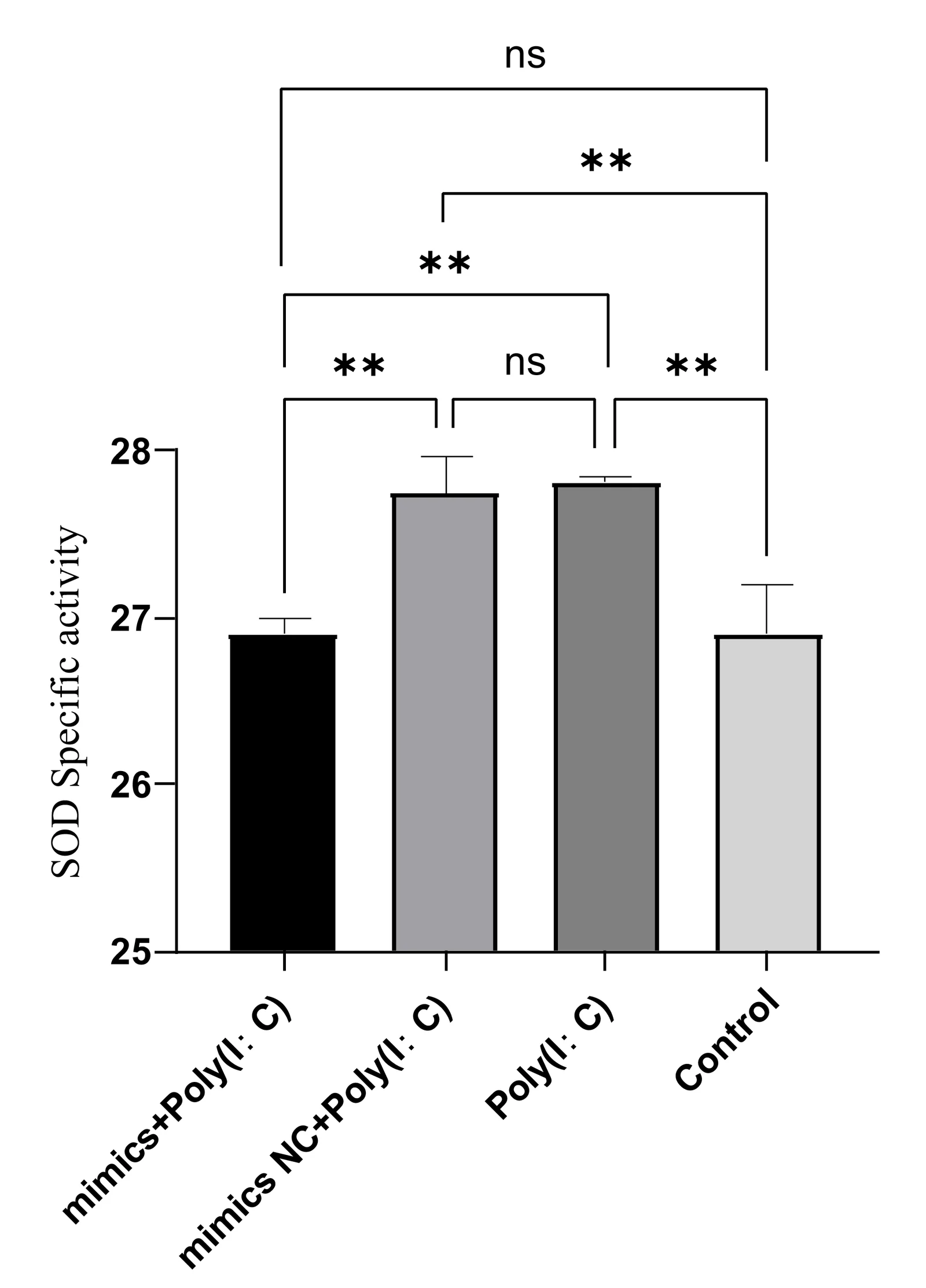
Effect of miR-122 on Poly(I:C)-induced SOD-specific activity. PCK cells were divided into a control group, a Poly(I:C) stimulation group, a miR-122 mimics negative control + Poly(I:C) co-treatment group, and a miR-122 mimics + Poly(I:C) co-treatment group. The experiments were performed using three independent biological replicates (*n* = 3). One-way ANOVA followed by Tukey’s multiple-comparisons test was used for the prespecified comparisons. **, *p* < 0.01; ns, not significant (*p* ≥ 0.05).

Poly(I:C) treatment increased cellular SOD activity. Co-treatment with miR-122 mimics significantly reduced SOD activity compared with the Poly(I:C)-only group. However, SOD activity in the miR-122 mimics + Poly(I:C) group was not significantly different from that in the blank control group. In contrast, the mimics NC did not significantly alter the Poly(I:C)-induced increase in SOD activity (Figure 4).

### 3.6. The Slc40a1 and Prkci Genes Are Target Genes of Mir-122

**Figure 5:**
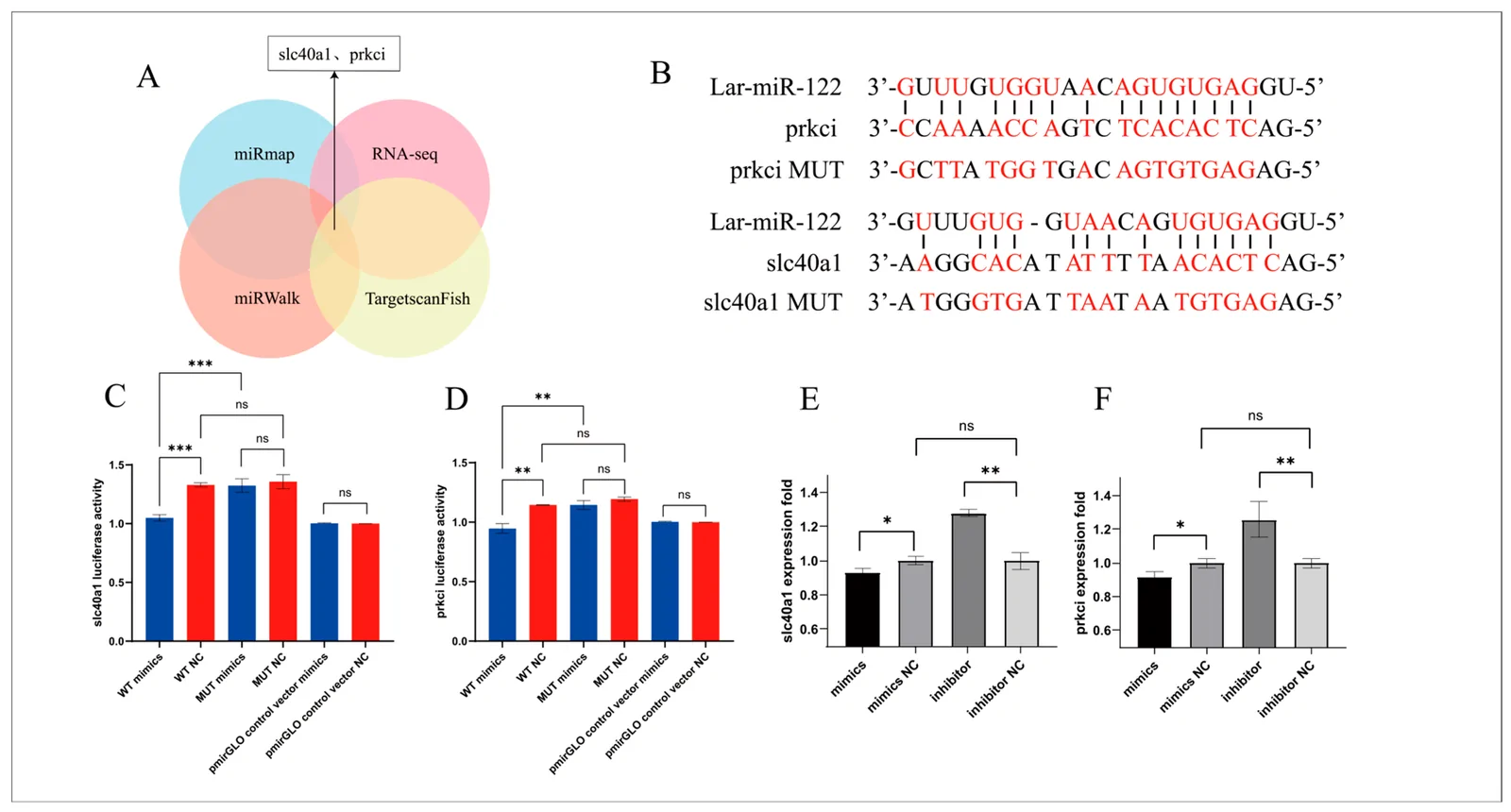
Prediction and validation of target genes in large yellow croaker. (**A**) Prediction of miR-122 target genes by integrating bioinformatics tools and RNA-seq data. (**B**) Identification of the predicted miR-122 binding sites and corresponding mutant sequences in the 3′-untranslated regions of protein kinase C iota (prkci) and solute carrier family 40 member 1 (slc40a1). Red nucleotides indicate the predicted miR-122 binding sites and the corresponding mutant sequence (**C**,**D**) Dual-luciferase reporter assays comparing WT, MUT, and empty pmirGLO constructs co-transfected with miR-122 mimics or mimics NC. Firefly luciferase activity was first normalized to Renilla luciferase activity. The resulting Firefly/Renilla ratios were then normalized to the mimics NC group of the empty pmirGLO vector, which was set to 1. Data were obtained from three independent co-transfection experiments. Two-way ANOVA followed by Tukey’s multiple-comparisons test was used for the prespecified comparisons. (**E**,**F**) Effects of miR-122 modulation on slc40a1 and prkci expression. The experiments were performed using three independent biological replicates (*n* = 3). One-way ANOVA followed by Tukey’s multiple-comparisons test was used for the prespecified comparisons. *, *p* < 0.05; **, *p* < 0.01; ***, *p* < 0.001; ns, not significant (*p* ≥ 0.05).

To uncover the direct effectors mediating the miR-122 phenotype, putative target genes of this microRNA were initially screened using a combination of bioinformatics platforms, including TargetScanFish 6.2, Miranda, and miRWalk. The resultant gene sets were subsequently overlapped with those exhibiting differential expression within the inhibitor group. Sequence analysis indicated that *slc40a1* and *prkci* each contain a highly evolutionarily conserved binding site for miR-122 within their respective 3′-untranslated regions (Figure 5A,B). To evaluate whether miR-122 directly interacts with the predicted binding sites in the 3′-UTRs of prkci and slc40a1, dual-luciferase reporter assays were conducted. Co-transfection of miR-122 mimics with the 3′-UTR constructs of *slc40a1* and *prkci* into HEK-293T cells resulted in significant reductions in luciferase activity for these two reporters. The same within-construct comparison was also performed for the MUT and empty pmirGLO vector controls. In addition, the WT and MUT constructs were compared under the mimics NC condition using Tukey-adjusted pairwise comparisons (Figure 5C,D). We next examined the mRNA transcript levels of *slc40a1* and *prkci* in the PCK cell line upon either enforced expression of miR-122 or its pharmacological inhibition. Notably, miR-122 overexpression led to decreased mRNA abundance for *slc40a1* and *prkci*. Conversely, treatment with a miR-122 inhibitor upregulated the mRNA levels of *slc40a1* and *prkci* genes in PCK cells (Figure 5E,F).

## 4. Discussion

The aquaculture industry for large yellow croaker is currently threatened by a wide range of diseases, which can be broadly divided into viral, bacterial, and parasitic infections. Viral diseases include iridovirus disease [29,30]. Bacterial diseases include visceral white spot disease caused by Pseudomonas plecoglossicida [31,32], vibriosis [33], and nocardiosis [34]. Parasitic diseases include cryptocaryoniasis [35], trypanosomiasis [36], scuticociliatosis [37], and benedeniasis, among others. Collectively, these diverse pathogens pose a persistent and serious challenge to the healthy and sustainable development of large yellow croaker aquaculture. To date, no single, definitive pathogen has been established as the causative agent of white gill disease in large yellow croaker, and its etiology remains unresolved.

This study systematically investigated the immunoregulatory role of miR-122 in large yellow croaker kidney cells (PCK cells) under poly(I:C) stimulation. Through transcriptome sequencing, bioinformatics prediction, and dual-luciferase reporter assays, we confirmed that *slc40a1* and *prkci* are direct target genes of miR-122. In addition, we found that miR-122 can regulate superoxide dismutase (SOD) activity in PCK cells, suggesting its possible involvement in the regulation of oxidative stress responses during antiviral immunity. These findings provide a new perspective for elucidating the molecular mechanisms by which miR-122 regulates innate immunity in teleost fish.

Previous studies have shown that poly(I:C) immune stimulation can induce an immune response in large yellow croaker [38], suggesting its involvement in the antiviral immune response of large yellow croaker. Poly(I:C) is a synthetic analog of viral double-stranded RNA, which can be recognized by pattern recognition receptors such as TLR3 and RIG-I-like receptors (RLRs) [39], subsequently initiating a signaling cascade and ultimately inducing the production of type I interferons and pro-inflammatory cytokines. To further explore the specific function and mechanism of miR-122 in the immune regulatory network of large yellow croaker, we transfected PCK cells with miR-122 mimics, mimics NC, miR-122 inhibitor, and inhibitor NC, respectively, and then performed transcriptome sequencing on cells from each group to investigate the effect of miR-122 functional perturbation on the expression profiles of immune-related genes. Our transcriptomic analysis further revealed that perturbation of miR-122 globally affects the expression of genes involved in ECM–receptor interaction, focal adhesion, and PPAR signaling pathways. Although these pathways are commonly associated with development and metabolism, their roles in immune cell migration, adhesion, and metabolic reprogramming during infection are increasingly recognized. Previous studies have demonstrated that miR-122 can exert antiviral effects through both direct interactions with viral RNA and regulation of host immune signaling. In hepatitis B virus (HBV) infection, miR-122 binds to a conserved sequence within the viral pregenomic RNA and inhibits viral gene expression and replication [40]. However, its effects are not uniformly antiviral. In hepatitis C virus (HCV) infection, miR-122 enhances viral replication, illustrating that its biological effects depend on the specific host–virus interaction [41]. Virus-promoting effects have also been reported in teleost fish. In miiuy croaker (Miichthys miiuy), miR-122 suppresses MAVS-mediated antiviral signaling and promotes Siniperca chuatsi rhabdovirus (SCRV) replication [42]. Similarly, in grouper (Epinephelus coioides), miR-122 overexpression enhances Singapore grouper iridovirus (SGIV) replication and reduces inflammatory cytokine expression [43]. In a separate poly(I)-stimulation study in miiuy croaker, miR-122 was shown to target DAK, a negative regulator of MDA5, indicating that it can also regulate RIG-I-like receptor signaling through a different host target [44]. Collectively, these studies highlight the importance of the viral context and downstream targets in determining the effects of miR-122. Our identification of prkci and slc40a1 as miR-122 targets extends the known regulatory network of this miRNA in teleost fish. Nevertheless, because the present study used poly(I) stimulation without measuring viral replication, whether these interactions ultimately restrict or facilitate viral infection remains to be established.

The dual-luciferase reporter assay results provide evidence for a sequence-specific interaction between miR-122 and the 3′-UTRs of prkci and slc40a1. In the WT reporter constructs, miR-122 mimics reduced normalized luciferase activity compared with the corresponding mimics NC. The same within-construct comparisons were also performed for the MUT constructs and the empty pmirGLO vector. In addition, the WT and MUT constructs were compared under the mimics NC condition to assess baseline differences in reporter activity. The preferential reduction observed in the WT constructs is consistent with the presence of functional miR-122-responsive sequences within the predicted binding regions, whereas mutation of these regions attenuated the reporter response. Together with the inverse changes in endogenous prkci and slc40a1 mRNA levels following miR-122 overexpression, these findings support a sequence-specific direct post-transcriptional interaction between miR-122 and the 3′-UTRs of prkci and slc40a1. However, the reporter assay alone cannot demonstrate physical binding, and further experiments are required to establish this interaction and its functional consequences more conclusively.

Among these, *prkci* was identified as a direct target of miR-122. *prkci* is a member of the atypical protein kinase C family and plays important roles in various cellular processes, including cell polarity, proliferation, and survival [45,46]. Importantly, *prkci* has been demonstrated to participate in the regulation of the NF-κB signaling pathway [47,48], which is a central pathway in antiviral and inflammatory responses [49]. For example, *PRKCI* can interact with the IKK complex to modulate NF-κB activation upon viral infection [50]. In this study, miR-122 overexpression significantly reduced the mRNA level of *prkci*, while miR-122 inhibition led to its upregulation, suggesting that miR-122-mediated suppression of *prkci* may contribute to fine-tuning NF-κB-driven inflammatory responses to avoid excessive immunopathology. This regulatory axis is consistent with the paradigm that miRNAs act as rheostats to balance pathogen clearance and immune homeostasis.

The second validated target gene, *slc40a1*, encodes the main cellular iron exporter at present [51]. Iron metabolism is closely linked to host immunity, because many pathogens, including viruses, require iron for replication, and the host restricts iron availability as a defense strategy [52], a process termed nutritional immunity [53]. In macrophages, ferroportin-mediated iron efflux is regulated by hepcidin and is critical for controlling intracellular pathogens [54,55]. In addition, intracellular iron levels affect the activity of iron-dependent enzymes, including ribonucleotide reductase, which is required for viral DNA synthesis [56]. In fish, the involvement of *slc40a1* in immune regulation is less studied, but our findings suggest a potentially conserved mechanism linking miRNA-mediated iron regulation to antiviral defense.

Our results showed an association between miR-122 modulation and SOD activity in PCK cells under poly(I:C) stimulation. Superoxide dismutase is a key antioxidant enzyme that catalyzes the dismutation of superoxide anions into hydrogen peroxide and molecular oxygen [57], thereby protecting cells from oxidative damage. Viral infections often induce oxidative stress, which can both promote viral replication and activate immune signaling pathways [58]. In this study, miR-122 overexpression reduced SOD activity relative to the Poly(I:C)-only group. However, because the miR-122 mimics + Poly(I:C) group was not significantly different from the blank control group, these results should be interpreted cautiously. The absence of a statistically significant difference does not demonstrate equivalence between the groups or exclude a biological effect of miR-122. Therefore, the present findings suggest a possible association between miR-122 and SOD activity, but they do not establish a definitive regulatory mechanism. This could be a consequence of targeting *slc40a1*, because iron is essential for the function of iron-sulfur cluster-containing enzymes and can catalyze the production of reactive oxygen species (ROS) via the Fenton reaction [59]. Alternatively, miR-122 may indirectly regulate SOD expression through signaling pathways such as Nrf2/ARE. The lower SOD activity observed after miR-122 overexpression is compatible with a change in the cellular redox response, but it does not establish a causal relationship between slc40a1 or prkci expression and SOD activity. Iron metabolism, ROS production, SOD isoform expression, and Nrf2/ARE signaling were not directly measured. Accordingly, links among miR-122, slc40a1, prkci, iron metabolism, Nrf2/ARE signaling, and SOD activity should be regarded as mechanistic hypotheses that require functional validation.

Several limitations should be considered. First, poly(I:C) models cellular recognition of viral double-stranded RNA but does not reproduce the complete viral life cycle; no viral load, replication, or infection-resistance outcome was measured. Second, the absence of unstimulated miR-122-modulation groups prevents attribution of the observed effects specifically to poly(I:C) stimulation. Third, the proposed links among miR-122, prkci, slc40a1, iron metabolism, Nrf2/ARE signaling, and SOD activity require targeted loss-of-function, gain-of-function, and rescue experiments. These limitations constrain the conclusions to miR-122-associated cellular responses under the tested in vitro conditions.

In summary, this study demonstrates that miR-122 in large yellow croaker acts as a negative regulator of immune-related cellular responses in PCK cells by targeting *prkci* and *slc40a1* and by modulating SOD-mediated oxidative stress. This work not only expands our understanding of the functional diversification of miR-122 in teleost immunity, but also provides potential molecular targets for breeding disease-resistant strains of large yellow croaker or for developing therapeutic strategies against viral outbreaks in aquaculture. Future studies should focus on dissecting the tissue-specific regulatory networks of miR-122 and evaluating its utility as a biomarker for viral disease susceptibility.

## 5. Conclusions

Under the poly(I:C)-stimulated in vitro conditions used here, miR-122 modulation was associated with broad transcriptional changes and with sequence-specific interactions involving the slc40a1 and prkci 3′-UTRs. Transcriptome analysis revealed that miR-122 overexpression and inhibition caused extensive changes in gene expression, with differentially expressed genes significantly enriched in pathways such as ECM–receptor interaction, focal adhesion, PPAR signaling, and TGF-β signaling. Reporter assays and reciprocal mRNA changes supported *slc40a1* and *prkci* as candidate direct targets of miR-122. miR-122 overexpression was also associated with lower poly(I:C)-induced SOD activity, although the relationship between these target genes and SOD activity was not established. These findings provide evidence for miR-122-associated cellular responses in PCK cells and identify hypotheses for further testing. This study provides new insights into miRNA-mediated immune regulation in teleosts and identifies potential molecular targets for disease-resistant breeding and antiviral prevention in large yellow croaker.

## Figures and Tables

**Table 1:** Sense and antisense sequences (5′→3′) of miR-122 mimics, NC, inhibitor, and inhibitor NC.

Group	Sequence (5′→3′)
miR-122 mimics sense	UGGAGUGUGACAAUGGUGUUUG
miR-122 mimics antisense	CAAACACCAUUGUCACACUCCAUUUG
miR-122 mimics NC sense	UUCUCCGAACGUGUCACGUTT
miR-122 mimics NC antisense	ACGUGACACGUUCGGAGAATT
miR-122 inhibitor sense	CAAACACCAUUGUCACACUCCA
miR-122 inhibitor NC antisense	CAGUACUUUUGUGUAGUACAA

**Table 2:** Sequences of primers employed in this study.

	Sequences of the Primers (5′-3)	
Gene	Forward Primer	Reverse Primer
atp11c	GAGATCCTGCTTGTGGTGTTC	GCTGCAGTCTGGATGATTGTAG
ptges	CGCCTTCACTTCCTGGTCTTCTTC	GGTATCTGTGCCACGGTGTAAGC
rmnd1	AGTCTTCATCTCTGCCTCGGTCAA	CCAGTACGCCAGGATCAGTCAAC
gcat	GTGCTGTCCGATGAGTTGAACCA	TGTGACTACGAGCCGCATACGA
itgav	GAACGGCTCGCTGCTCTACATC	GTCTCTTGACTCCAGGTCTCTTCTCT
cnpy2	CAGCAAGACCAGGACAGGACAT	CCCGTCTGGATCATCTTCTTTGGA
slc40a1	GCCTGCTGGTCATCATCTCTGTC	TGTCCACCGTCTCCTCCTTCTG
prkci	GCCATCTGTGCGGATCGTATCTG	GCATCATTGGTTCCTGGATCATCTGT
p4ha1	GACCTGGAGAGCCTTGTA	GTCTGTAAGTGTCTTGTAACC
aktip	CCTTCATCATCCGCTGTC	CCATCTCCTCCTCGTTGA

**Table 3:** (**A**) Differentially expressed immune-related genes in the mimics group. (**B**) Differentially expressed immune-related genes in the inhibitor group.

**(A)**		
**Gene ID**	**Gene Description**	**Log_2_FC** **(Mimics vs** **.** **Mimics NC)**
gene-atp11c	ATPase phospholipid transporting 11C, transcript variant X1	−0.28528985529332
gene-ptges	prostaglandin E synthase	0.41270941281526
gene-rmnd1	required for meiotic nuclear division 1 homolog	0.51368606811067
gene-gcat	glycine C-acetyltransferase	0.56963981466433
gene-itgav	integrin subunit alpha V	0.34672560850192
gene-cnpy2	canopy FGF signaling regulator 2	0.54429233394707
gene-slc40a1	solute carrier family 40 member 1	−0.53356140486502
gene-prkci	protein kinase C iota	−0.55359877669845
**(B)**		
**Gene ID**	**Gene Description**	**Log_2_FC** **(Inhibitor vs** **.** **Inhibitor NC)**
gene-atp11c	ATPase phospholipid transporting 11C, transcript variant X1	0.25311460295796
gene-ptges	prostaglandin E synthase	−0.40919216130314
gene-rmnd1	required for meiotic nuclear division 1 homolog	0.47801434714366
gene-gcat	glycine C-acetyltransferase	0.34954275502261
gene-itgav	integrin subunit alpha V	0.29789918538185
gene-cnpy2	canopy FGF signaling regulator 2	0.55091711396801
gene-p4ha1	prolyl 4-hydroxylase subunit alpha 1, transcript variant X2	0.87264213683532
gene-aktip	AKT interacting protein, transcript variant X1	−0.3942642772679

## Data Availability

The raw RNA-seq data generated in this study are available in the NCBI Sequence Read Archive under accession number PRJNA1501314. The supplementary datasets, including Tables S1–S7, will be made available through the journal’s Supplementary Materials webpage upon publication. All data for this study are presented in the main text and appendices.

## Supplementary Materials

The following supporting information can be downloaded at https://www.mdpi.com/article/10.3390/ani16213489/s1. Table S1: Summary of sequencing data quality results. Table S2: Differentially expressed genes between the miR-122 mimics and mimics NC groups. Table S3: Differentially expressed genes between the miR-122 inhibitor and inhibitor NC groups. Table S4: GO enrichment analysis of differentially expressed genes between the miR-122 mimics and mimics NC groups. Table S5: GO enrichment analysis of differentially expressed genes between the miR-122 inhibitor and inhibitor NC groups. Table S6: KEGG pathway enrichment analysis of differentially expressed genes between the miR-122 mimics and mimics NC groups. Table S7: KEGG pathway enrichment analysis of differentially expressed genes between the miR-122 inhibitor and inhibitor NC groups.
