# Supplementary material for: The Regulatory Mechanism of MiR-122 in the Antiviral Immunity of Larimichthys crocea Kidney Cells
Source: Animals (Basel). 2026 Nov 4;16(21):3489. doi: 10.3390/ani16213489 (PMC13761911; doi:10.3390/ani16213489)
Supplement: Supplementary file 1 [file animals-16-03489-s001.zip › Table S1.pdf]

Table S1
Summary of sequencing data quality results.

Sample	Raw reads	Raw bases	Clean reads	Clean bases	Error rate(%)	Q20(%)	Q30(%)	GC content(%)
mimics_P_1	37104772	5602820572	36804766	5535275491	0.0128	98.86	94.47	49.46
mimics_P_2	37185386	5614993286	36879670	5546238962	0.0127	98.89	94.61	49.63
mimics_P_3	37051866	5594831766	36748540	5526609064	0.0128	98.85	94.46	49.34
inhibitor_P_1	37672220	5688505220	37358262	5618209145	0.0127	98.88	94.64	49.5
inhibitor_P_2	39553578	5972590278	39226032	5896846242	0.0127	98.87	94.59	49.44
inhibitor_P_3	40854398	6169014098	40478864	6085513786	0.0127	98.86	94.62	49.49
mimicsNC_P_1	36661014	5535813114	36326942	5462106630	0.0129	98.78	94.27	50.53
mimicsNC_P_2	44908582	6781195882	44492532	6690135140	0.0127	98.84	94.58	49.86
mimicsNC_P_3	41415432	6253730232	41065466	6172225107	0.0127	98.89	94.66	49.52
inhibitorNC_P_1	39650526	5987229426	39305662	5912666474	0.0127	98.87	94.59	49.24
inhibitorNC_P_2	49047334	7406147434	48599844	7308023757	0.0127	98.86	94.62	49.36
inhibitorNC_P_3	41114998	6208364698	40785064	6133123110	0.0127	98.87	94.56	49.61
